# Efficacy of acupuncture on acute pharynx infections: A systematic review and meta-analysis

**DOI:** 10.1097/MD.0000000000034124

**Published:** 2023-06-23

**Authors:** Shuo Zhang, Yang Cui, Xinyu Zhou, Delong Wang, Jiantao Yin, Xiangyue Meng, Yu Cao, Quan Li, Hongna Yin

**Affiliations:** a Heilongjiang University of Chinese Medicine, Harbin, China; b The Second Affiliated Hospital of Heilongjiang University of Chinese Medicine, Harbin, China.

**Keywords:** acupuncture, acupuncture analgesia, acute pharynx infections, meta-analysis, systematic review

## Abstract

Several clinical reports have focused on acupuncture for the treatment of acute pharyngeal infections. However, the efficacy and safety of acupuncture for the treatment of acute pharyngeal infections are controversial. To systematically assess the efficacy and safety of acupuncture in treating acute pharynx infections, thus providing a reference for clinical decision-making.

**Methods:**

We searched PubMed, CENTRAL, Embase, Web of Science, China National Knowledge Infrastructure, China Biomedical, clinical research registration platforms, gray literature, and reference lists of the selected studies from inception to October 30, 2022. The risk of bias assessment was performed using RevMan. The meta-analysis was performed using STATA with the Hedges’ *g* value. We also performed a subgroup analysis, meta-regression, and publication bias detection using Harbord’s and Egger’s tests.

**Results:**

We included 19 randomized controlled trials comprising 1701 patients, of which only one study had a high risk of bias. The primary outcome, i.e., the response rate, revealed that acupuncture was more effective than antibiotics. The secondary results revealed that the differences in the reduction of VAS scores, sore throat duration, and white blood cell counts were statistically significant in the acupuncture group compared with the antibiotic group. However, the difference in the modulation of the neutrophil percentage and C-reactive protein levels was insignificant. Moreover, the acupuncture treatment resulted in a lower incidence of adverse events than the antibiotic treatment.

**Conclusions:**

Thus, acupuncture therapy for acute pharyngeal infections is safe and its response rate is superior to that of antibiotics. Acupuncture showed positive outcomes for alleviating the sore throat symptoms, shortening the sore throat duration, and improving the immune inflammation index. Nevertheless, owing to the limitations of this study, our conclusions should be interpreted with caution. More high-quality trials are warranted in the future for improving the methodology and reporting quality.

## 1. Introduction

Acute pharyngeal infections are characterized by discomfort, pain, or itchiness in the throat attributed to various infectious causes, including acute pharyngitis, tonsillitis, and retropharyngeal abscesses.^[1]^ In many pharyngeal infections, the leading causes are self-limiting viral infections (40–80%), with group A streptococcal infections being the most common (15–30%); a proportion of immunocompromised patients may be affected by fungal infections.^[1]^ Despite the wide range of infectious agents, acute pharyngitis is predominantly self-limiting and rarely produces significant sequelae.^[2]^ The most prominent symptom is sore throat or pain upon swallowing and fever; other symptoms and signs may include headache, gastrointestinal discomfort, tonsillopharyngeal erythema, and tonsillopharyngeal exudate.^[3]^ Not all signs and symptoms may be present, and many cases could be relatively mild with no exudates.

The incidence of acute pharyngeal infections is high worldwide, accounting for more than 2% and 5% of adult and pediatric populations, respectively,^[4]^ estimating to approximately 11 and 18 million individuals, respectively annually seeking treatment for pharyngitis.^[5]^ However, the actual number of individuals is much higher, since more than 4 to 6 times greater number of individuals with sore throats do not seek treatment.^[6]^ Seasonal variability is a common risk factor for acute pharyngeal infections attributed to respiratory viruses and group A streptococcal. Individuals are more susceptible to pharyngitis in winter and early spring.^[1]^ Exposure is the leading risk factor for acute pharyngeal infections.^[7]^ Even though the underlying cause is generally benign, and the course is characteristically mild, the high incidence of acute pharyngitis seriously impacts public health, decreasing productivity, resulting in economic burden, and triggering antibiotic resistance.^[8,9]^

Unfortunately, the management of acute pharyngeal infections remains challenging. Clinical treatment of pharyngitis can be divided into symptomatic and antimicrobial therapy.^[4]^ Symptomatic treatments include nonsteroidal anti-inflammatory drugs, paracetamol, laryngeal preparations (local anesthetics and nonsteroidal anti-inflammatory drugs nonsteroidal anti-inflammatory drugs), oral corticosteroids, etc.^[2]^ The primary antimicrobial treatment is t antibiotic administration, of which penicillin remains the first choice,^[10,11]^ with amoxicillin, cefadroxil, cephalexin, and azithromycin serving as alternatives for patients who are intolerant to penicillin.^[1]^ Nevertheless, some drugs used for symptomatic treatment can cause serious adverse effects with a low probability, such as throat preparations^[12–14]^ and diclofenac,^[15]^ or have limited efficacy, such as oral corticosteroids^[2]^ and paracetamol.^[16]^ Moreover, unnecessary antibiotic prescriptions pose additional risks to patients and promote the development of drug resistance.^[17]^ Patient expectations are not antibiotic action but pain relief.^[7]^ Therefore, identifying an effective treatment for relieving the pain associated with acute pharyngitis with fewer side effects is crucial.

Acupuncture, a non-drug therapy, is valuable for pain management,^[18]^ with immediate effects and minimal adverse reactions.^[19]^ With its increasing use in clinical settings, acupuncture has been widely used to treat acute pharyngeal infections. A 1973 randomized controlled trial (RCT) demonstrated that acupuncture could relieve sore throats better than sham acupuncture.^[20]^ Another RCT in 2015 demonstrated that acupuncture reduced sore throat within 24 hours compared to conventional treatment.^[21]^ However, the available evidence from published studies has reported conflicting results. A 2009 RCT revealed no significant efficacy of acupuncture for acute pharyngeal infections compared to sham acupuncture.^[22]^ Additionally, an international guideline suggested insufficient evidence supporting the effectiveness of acupuncture for acute sore throats.^[23]^ Consequently, these heterogeneous results make determining the efficacy of acupuncture in the treatment of acute pharyngeal infections challenging, thereby confounding clinical decision-making.

This meta-analysis provides an evidence-based medical basis for acupuncture therapy for acute pharyngeal infections and guides clinical decision-making to address the abovementioned inconsistencies.

## 2. Methods

This study was strictly implemented according to the Preferred Reporting Items for Systematic Reviews and Meta-Analyses^[24]^ and registered with PROSPERO (CRD42022372644). Since our article is a meta-analysis based on previously published studies, no ethical approval or patient consent was required.

### 2.1. Data sources

We searched 6 electronic databases, including PubMed, Embase, Cochrane Library, Web of Science, China National Knowledge Infrastructure, and China Biomedical, from inception to October 25, 2022; the language was restricted to English and Chinese.

English search terms included: “Pharyngitis,” “Pharyngitides,” “Sore Throats,” “Throat, Sore,” “Sore Throat,” “Tonsillitis,” “Acupuncture Therapy,” “Acupuncture Analgesia,” “Acupuncture, Ear,” “Electroacupuncture,” “Meridians,” “Acupuncture Points,” “Moxibustion,” “acupuncture,” “manual acupuncture,” “surrounding acupuncture,” “surround needling,” “auricular acupuncture,” “ear acupuncture,” “electro-acupuncture,” “fire needling,” “fire needle,” “warm needling,” “warm needle,” “thermal moxibustion,” “medicated thread moxibustion,” “pricking and cupping,” “pricking blood and cupping,” “blood-letting puncture and cupping,” “Fu’s subcutaneous needling,” “Fu’s acupuncture,” “acupoint catgut embedding,” “catgut embedding,” “dry needling,” “dry needle,” and “laser acupuncture.”

Chinese search terms included: “咽炎,” “扁桃体炎,” “乳蛾,” “喉痹,” “咽喉痛,” “咽痛,” “针,” “刺,” “放血,” “灸,” “穴,” “埋线,” “拔罐.”

The complete search strategy used for the Cochrane Central Register of Controlled Trials and other databases is shown in Table 1.

**Table 1:** CENTRAL: search strategy.

Query	Search terms	Results	Date
*#1*	MeSH descriptor: [Pharyngitis] explode all trees	1378	Oct. 25 2022
*#2*	MeSH descriptor: [Tonsillitis] explode all trees	426	Oct. 25 2022
*#3*	(Pharyngitis):ti, ab, kw	2563	Oct. 25 2022
*#4*	(Pharyngitides):ti,ab,kw	0	Oct. 25 2022
*#5*	(Sore Throats):ti,ab,kw	84	Oct. 25 2022
*#6*	(Throat, Sore):ti,ab,kw	3784	Oct. 25 2022
*#7*	(Sore Throat):ti,ab,kw	3784	Oct. 25 2022
*#8*	(Tonsillitis):ti,ab,kw	1155	Oct. 25 2022
*#9*	*#1 OR #2 OR #3 OR #4 OR #5 OR #6 OR #7 OR #8*	6451	Oct. 25 2022
*#10*	MeSH descriptor: [Acupuncture Therapy] explode all trees	5354	Oct. 25 2022
*#11*	MeSH descriptor: [Acupuncture Analgesia] explode all trees	302	Oct. 25 2022
*#12*	MeSH descriptor: [Acupuncture, Ear] explode all trees	222	Oct. 25 2022
*#13*	MeSH descriptor: [Electroacupuncture] explode all trees	897	Oct. 25 2022
*#14*	MeSH descriptor: [Meridians] explode all trees	2321	Oct. 25 2022
*#15*	MeSH descriptor: [Acupuncture Points] explode all trees	2276	Oct. 25 2022
*#16*	MeSH descriptor: [Moxibustion] explode all trees	525	Oct. 25 2022
*#17*	MeSH descriptor: [Acupuncture] explode all trees	166	Oct. 25 2022
*#18*	(Acupuncture):ti,ab,kw	17320	Oct. 25 2022
*#19*	(Acupuncture Therapy):ti,ab,kw	9454	Oct. 25 2022
*#20*	(Acupuncture Analgesia):ti,ab,kw	1192	Oct. 25 2022
*#21*	(Acupuncture, Ear):ti,ab,kw	740	Oct. 25 2022
*#22*	(Electroacupuncture):ti,ab,kw	3069	Oct. 25 2022
*#23*	(Meridians):ti,ab,kw	469	Oct. 25 2022
*#24*	(Acupuncture Points):ti,ab,kw	5130	Oct. 25 2022
*#25*	(Moxibustion):ti,ab,kw	2200	Oct. 25 2022
*#26*	(manual acupuncture):ti,ab,kw	654	Oct. 25 2022
*#27*	(surrounding acupuncture):ti,ab,kw	63	Oct. 25 2022
*#28*	(surround needling):ti,ab,kw	12	Oct. 25 2022
*#29*	(auricular acupuncture):ti,ab,kw	839	Oct. 25 2022
*#30*	(ear acupuncture):ti,ab,kw	740	Oct. 25 2022
*#31*	(electro-acupuncture):ti,ab,kw	656	Oct. 25 2022
*#32*	(fire needling):ti,ab,kw	56	Oct. 25 2022
*#33*	(fire needle):ti,ab,kw	129	Oct. 25 2022
*#34*	(warm needling):ti,ab,kw	111	Oct. 25 2022
*#35*	(warm needle):ti,ab,kw	134	Oct. 25 2022
*#36*	(thermal moxibustion):ti,ab,kw	41	Oct. 25 2022
*#37*	(medicated thread moxibustion):ti,ab,kw	13	Oct. 25 2022
*#38*	(pricking and cupping):ti,ab,kw	68	Oct. 25 2022
*#39*	(pricking blood and cupping):ti,ab,kw	21	Oct. 25 2022
*#40*	(blood-letting puncture and cupping):ti,ab,kw	26	Oct. 25 2022
*#41*	(Fu’s subcutaneous needling):ti,ab,kw	30	Oct. 25 2022
*#42*	(Fu’s acupuncture):ti,ab,kw	50	Oct. 25 2022
*#43*	(acupoint catgut embedding):ti,ab,kw	155	Oct. 25 2022
*#44*	(catgut embedding):ti,ab,kw	199	Oct. 25 2022
*#45*	(dry needling):ti,ab,kw	897	Oct. 25 2022
*#46*	(dry needle):ti,ab,kw	408	Oct. 25 2022
*#47*	(laser acupuncture):ti,ab,kw	666	Oct. 25 2022
*#48*	*#10 OR #11 OR #12 OR #13 OR #14 OR #15 OR #16 OR #17 OR #18 OR #19 OR #20 OR #21 OR #22 OR #23 OR #24 OR #25 OR #26 OR #27 OR #28 OR #29 OR #30 OR #31 OR #32 OR #33 OR #34 OR #35 OR #36 OR #37 OR #38 OR #39 OR #40 OR #41 OR #42 OR #43 OR #44 OR #45 OR #46 OR #47*	20506	Oct. 25 2022
*#49*	*#9 AND #48*	50	Oct. 25 2022

ab = abstract, kw = keywords, ti = title.

In addition, we searched the World Health Organization International Clinical Trial Registration platform (https://trialsearch.who.int), China Clinical Trial Registration Center (https://www.chictr.org.cn), previous meta-analyses, and gray literature (http://www.opengrey.eu).

Two researchers crosschecked the search results and searched the reference lists of the included studies.

### 2.2. Inclusion and exclusion criteria

#### 2.2.1. Inclusion criteria methods.

The following inclusion criteria were adopted based on the PICOS (population, intervention, comparison, outcomes, Studies) process.

##### 2.2.1.1. Population.

The diagnosis of acute pharyngeal infections was supported by the patient’s medical history and physical examination.^[25]^ Patients with sore throat as the primary symptom, with or without hyperpyrexia, were included regardless of the age, sex, or race.

##### 2.2.1.2. Treatment group.

Acupuncture, alone or in combination with antibiotic treatment, was included. In this study, acupuncture techniques were defined as manual acupuncture, electroacupuncture, acupuncture bloodletting, and other acupuncture techniques used in clinical practice (e.g., fire and warm needling); however, acupoint injections were not included. No limitations were imposed on the needle material, acupoint matching, needle retention duration, or treatment course.

##### 2.2.1.3. Control group.

The control group received sham acupuncture, no treatment, or antibiotics. Antibiotics were limited to those with clear evidence of their effectiveness against pharyngitis and tonsillitis (including penicillin, amoxicillin, and cephalosporin) to ensure homogeneity. When a combination of antibiotics was administered, the same antibiotics were administered to the treatment and control groups.

##### 2.2.1.4. Outcome indicators.

1. The primary outcome was the clinical response rate of acute pharyngeal infections.

Referring to the diagnostic efficacy standard of Traditional Chinese Medicine Disease and Syndrome: Cure: the disappearance of clinical symptoms and signs, and reduction of symptom scores by ≥95%; Markedly effective: significant improvement of symptoms and signs, and reduction of symptom scores by ≥70%; Effective: reduction of total scores of symptoms and signs by ≥30%; and Ineffectiveness: no significant improvement of symptoms and signs, or reduction of scores by less than 30%.


$$\text{Clinical }\text{​}\text{​}\;\text{​}\text{​}\text{ response }\text{​}\text{​}\;\text{​}\text{​}\text{ rate}=\frac{\left(\text{cured}+\text{markedly }\text{​}\text{​}\;\text{​}\text{​}\text{ effective}+\text{effective}\right)\;\text{​}\text{​}\;\text{​}\text{​}\text{ cases}}{\text{the }\text{​}\text{​}\;\text{​}\text{​}\text{ total }\text{​}\text{​}\;\text{​}\text{​}\text{ cases}}\text{*}100\;\text{​}\text{​}\%\text{​}$$

(b) The secondary outcomes were improvements in the following areas:
  1. VAS scores
  2. Sore throat duration
  3. Laboratory tests
  4. Adverse events (AEs)

##### 2.2.1.5. Study design.

Studies based on needle insertion or acupoint stimulation for therapeutic purposes, including RCTs that evaluated the efficacy of acupuncture for acute pharyngeal infections, were included.

#### 2.2.2. Exclusion criteria.

Studies were excluded for any of the following factors.

1. Secondary analysis or duplicate publications (in multilingual publications, only the earliest publication was chosen).
2. Full text not available after contacting the corresponding author.
3. Flawed study design, such as not strictly following the principles of randomization (e.g., randomization performed based on the order in which patients were admitted to the hospital, odd and even case numbers, or birth dates).
4. Missing baseline data.
5. Outcome indicators were not available.
6. The type of intervention included drug treatment with no evidence-based efficacy (e.g., Chinese herbal medicine).^[23,26]^

### 2.3. Trial selection

After using EndNote X9 (Clarivate Analytics, London, UK) to delete duplicate records, 2 researchers independently reviewed all the titles and abstracts based on the inclusion and exclusion criteria; studies that did not meet the inclusion criteria were excluded. Subsequently, the full text of the studies that met the requirements was carefully reviewed to determine whether it should be included in the analysis. Differences in opinion were resolved through discussion. If an agreement could not be reached, the decision arbitrator was consulted. All the researchers were medical practitioners with more than 2 years of clinical experience in acupuncture.

### 2.4. Data extraction

A fixed protocol was used to extract the following information. After the final selection of the included studies, 2 investigators independently extracted the data and created summary tables. Data were extracted based on the model, including the study (first author, publication year, and location), participant characteristics (patient source, sample size, and mean age), intervention (type, acupoint, specific drug, dose, and frequency, and treatment course), and outcome indicators.

### 2.5. Risk of bias assessment

According to the Cochrane Collaboration recommendation,^[27]^ 2 researchers independently assessed the following 7 major risks of bias for all the included studies using RevMan version 5.4 (The Cochrane Collaboration, London, England): random sequence generation, allocation hiding, blinding of participating researchers, blinding of outcome evaluation, incomplete outcome data, selective reporting, other types of bias, and outcomes classified into 3 grades (high, low, and unclear).

### 2.6. Data synthesis and analysis

The meta-analysis was performed using Stata 15.1 software (StataCorp LLC, College Station, TX). Continuous variables were assessed using standardized mean differences (Hedges’ *g*) and corresponding 95% confidence intervals (CIs) for the effectiveness analysis of statistics; a difference of <.05 was considered statistically significant. Dichotomous variables are expressed as relative risk and corresponding 95% CIs. An odds ratio (OR) > 1 indicated high efficacy in the treatment group, an OR < 1 indicated high efficacy in the control group, and OR = 1 indicated that the difference between the 2 groups was not statistically significant.

Heterogeneity was assessed using the *I*^2^ test. If *I*^2^ was ≤ 50%, the heterogeneity was insignificant and the fixed-effects model was used; *I*^2^ > 50% indicated substantial heterogeneity and a random-effects model was used. If an outcome indicator contained more than 9 studies, meta-regression analysis was used to clarify the source of heterogeneity among the studies; otherwise, subgroup and sensitivity analyses were performed. Subgroup analyses were performed to investigate the effects of other potentially confounding variables. A sensitivity analysis was performed by eliminating each data point to evaluate the stability of the results. Harbord’s test was used to quantify the significance of publication bias for dichotomous variables, which maintains the power of Egger’s test while reducing the false positive rate.^[28]^ Egger’s test was used to quantify the significance of publication bias for continuous variables (with *P* < .05, considered significant). If the outcome was presented as the mean and standard error (SE), the standard deviation (SD) was calculated as SD = SE × $\sqrt{n}$ (n = sample size).^[27]^

## 3. Results

### 3.1. Literature retrieval

**Figure 1.**
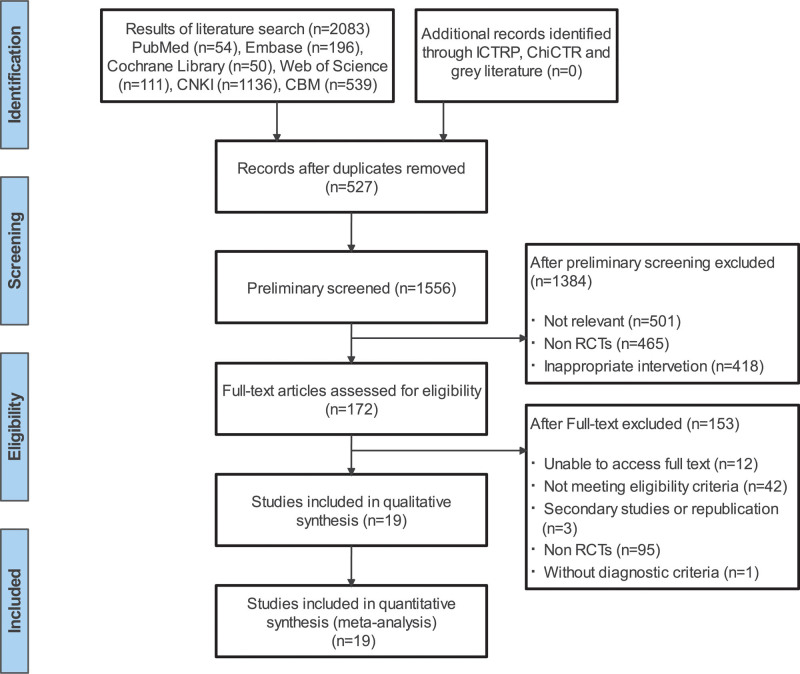
Flow chart of study selection. RCT = randomized controlled trial.

The initial search resulted in 2086 pending records, and 172 potentially eligible articles were selected for full-text review after eliminating duplicates and reading the titles and abstracts. Among these 172 articles, twelve complete reports could not be obtained, forty-two did not meet the inclusion criteria, 3 were republished, ninety-five were not RCTs, and one did not have clear diagnostic criteria (Fig. 1). Finally, 19 RCTs were included in the meta-analysis.^[21,22,29–45]^

### 3.2. Study characteristics

Table 2 shows that all the included studies were published between 2006 and 2022. Seventeen studies were conducted in China,^[29–45]^ and 2 were conducted in the United States^[21]^ and Germany.^[22]^ The types of acupuncture include blood-letting puncture,^[29–32,34–37,39,41–45]^ manual acupuncture,^[22,33,38,40]^ and auricular acupuncture.^[21]^ Eight studies used acupuncture plus antibiotics, the same as the control group.^[32,34–36,39,41–43]^ Four studies reported AEs to antibiotics,^[34,36,38,45]^ including nausea, vomiting, rash, and allergy. Notably, no studies reported AEs in acupuncture interventions.

**Table 2:** Characteristics of randomized controlled trials included in the meta-analysis.

Study	Country	Participant characteristics		Treatment group		Control group		Period	Outcome
		Age, mean (SD)	N (TG/CG)	Type	Acupoint and frequency	Type	Usage and dosage		
Johannes, et al (2009)	Germany	TG:32.50 CG:27.60	60 (30/30)	Manual acupuncture	LI 8, LI 10; once	Sham acupuncture	/	15 min	②
David, et al (2015)	USA	TG:34.00 CG:31.00	54 (27/27)	Auricular acupuncture	Cingulate gyrus, thalamus, omega 2, point zero, and *shenmen*; once	Antibiotics	/	15 min	②
Yang, et al (2012)	China	TG:28.56 (15.43) CG:29.16 (12.21)	74 (36/38)	Manual acupuncture	LI4; once	Sham acupuncture	/	1 min	②
Liao, et al (2012)	China	TG:26.40 CG:24.20	60 (30/30)	Blood-letting	Throat, helix, and *sanshang*; qd	Penicillin sodium	0.48 g; bid, im	7 d	①
Shen, et al (2013)	China	TG:4.00 (2.00) CG:4.00 (2.00) CG:4.00 (2.00)	25 (25/25/25)	Blood-letting plus Penicillin sodium	LI11, LI4, DU14, LU11, Ears tips; qd	Blood-letting/Penicillin sodium	60 mg/kg; bid, im	3 d	①
	China	TG:4.00 (2.00) CG:4.00 (2.00) CG:4.00 (2.00)	25 (25/25/25)	Blood-letting plus Penicillin sodium	LI11, LI4, DU14, LU11, Ears tips; qd	Blood-letting/Penicillin sodium	60 mg/kg; bid, im	5 d	①
Chen, et al (2006)	China	TG:36.75 (9.59) CG:36.36 (10.56)	120 (60/60)	Blood-letting	Throat, helix, and *sanshang*; qd	Penicillin sodium	0.48 g; bid, im	7 d	①②④
Ai, et al (2020)	China	TG:24.00 (5.00) CG:22.00 (5.00)	60 (30/30)	Fire needle bloodletting plus amoxicillin	LI20; qod	Amoxicillin	0.375 g; tid, po	5 d	①③④
Ma, et al (2019)	China	TG:5.48 (1.29) CG:5.48 (1.29)	86 (43/43)	Blood-letting plus cefathiamidine	LI11, LI4, Ears tips; qd	Cefathiamidine	2–5 g/d; iv	5 d	①②
Peng, (2016)	China	TG:35 CG:35	58 (30/28)	Blood-letting	Throat; once	Cefdinir Capsules	0.1 g; tid, po	5 d	①
Chen, (2018)	China	TG:35.33 CG:35.64	80 (40/40)	Blood-letting	Lu11, DU14, BL13; qd	Cefmetazole sodium	2 g; bid, iv	3 d	①③④
Chen, (2017)	China	TG:22.60 (1.48) CG:22.51 (1.52)	60 (30/30)	Blood-letting plus Penicillin sodium	LU11; qd	Penicillin sodium	1.44 g; bid, iv	5 d	①③
Zhang, et al (2017)	China	TG:28.75 CG:27.89	80 (40/40)	Manual acupuncture	LU11, LI4, ST37, ST40; qd	Cefuroxime Axetil	0.25 g; bid, po	7 d	①
Hu, (2006)	China	TG:27.25 (2.20) CG:26.8 (2.70)	134 (92/42)	Manual acupuncture	*Shesanzhen* point, LI11, LU5, LI4, ST40, ST44, LU10, KI6, SP6; qd	Penicillin sodium	0.48 g; bid, im	3 d	①
Wang, (2020)	China	TG:29.18 (7.98) CG:28.36 (8.05)	74 (37/37)	Blood-letting plus cefathiamidine	Ears tips; qd	Cefathiamidine	50 mg/kg; bid, iv	7 d	①
Meng, et al (2022)	China	TG:5.02 (0.98) CG:5.07 (1.02)	60 (30/30)	Blood-letting	Ears tips, LU11, LI1; qd	Penicillin sodium	0.48 g; bid, im	3 d	①③
Xu, et al (2015)	China	TG:6.03 (1.96) CG:6.10 (2.07)	120 (60/60)	Blood-letting plus amoxicillin	PC9; once	Sham acupuncture plus amoxicillin	0.375 g; tid, po	3 d	①④
Wang, et al (2018)	China	TG:4.41 (1.55) CG:4.36 (1.55)	52 (26/26)	Blood-letting plus cefathiamidine	LI11, LI4, DU14; qd	Cefathiamidine	50 mg/kg; bid, iv	7 d	①④
Xiao, et al (2006)	China	TG:42.60 CG:41.30	80 (40/40)	Blood-letting	Throat, helix, and *sanshang*; qd	Penicillin sodium	0.48 g; bid, im	7 d	①
Yao, et al (2018)	China	TG:6.34 (2.62) CG:6.00 (2,46)	63 (32/31)	Blood-letting plus cefaclor	EX-UE10; qd	Cefaclor for suspension	20 mg/kg; qd, po	3 d	①②

CG = control group, SD = standard deviation, TG = treatment group.

① = the clinical response rate; ② = change from baseline VAS scores; ③ = throat sore time; ④= Laboratory indicators.

### 3.3. Study quality

**Figure 2.**
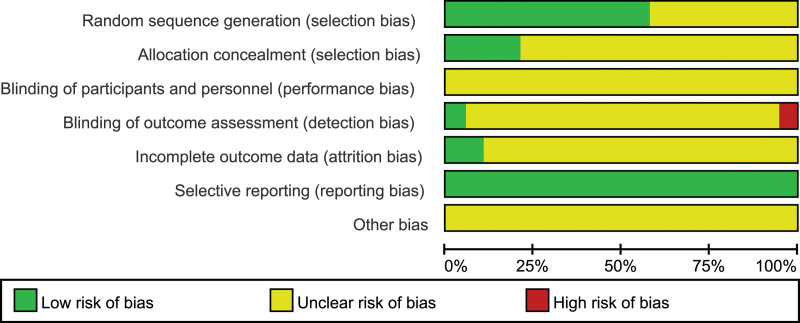
Assessment of risk of bias with selected studies.

Eleven studies mentioned random sequence generation^[21,22,31,32,34,35,39–42,45]^; 10 studies used the random number table method,^[21,22,31,32,35,39–42,45]^ and one used a central randomization system,^[34]^ while 8 articles did not specifically describe the sequence generation method.^[29,30,33,36–38,43,44]^ Three studies used opaque, sealed envelopes for allocation concealment.^[22,40,42]^ One study^[34]^ used the central randomization system method for allocation concealment, whereas the remaining studies did not describe the concealment distribution method. Considering result evaluator blindness, 2 studies described the concrete process,^[21,40]^ whereas the remaining 17 studies did not mention whether the evaluator was blinded or designed. Two studies reported the data and reasons for loss to follow-up.^[21,22]^ All 19 studies reported the outcome indicator results. No other biases were explicit in any of the studies. Regarding the quality assessment of the studies, it was difficult to camouflage and hide acupuncture therapy from blinded physicians and patients owing to its peculiarity. Figure 2 summarizes the quality assessment and risk of bias of all the studies.

### 3.4. Meta-analysis findings

#### 3.4.1. Clinical response rate.

**Figure 3.**
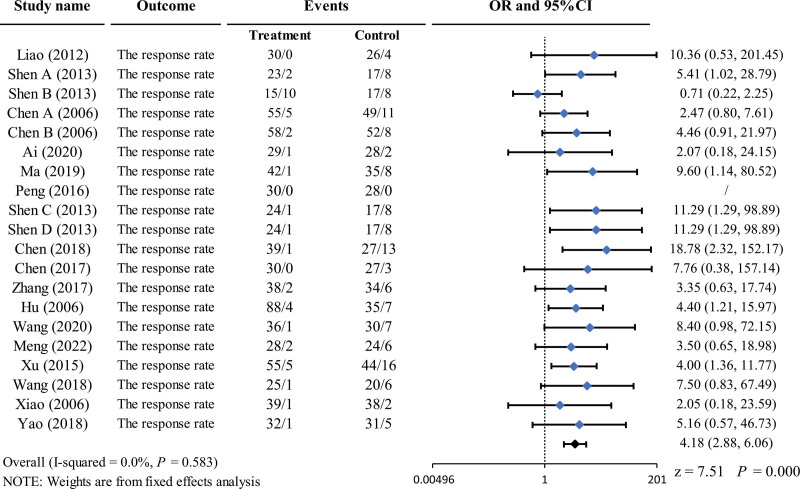
Forest plot of the response rate. CIs = confidence intervals, OR = odd ratio.

Sixteen studies^[29–39,41–45]^ reported a response rate of 1513 patients. The heterogeneity between the studies was *I*^2^ = 0.0%. Therefore, we selected a fixed-effects model for the meta-analysis. The results demonstrated that acupuncture therapy was superior to antibiotics for acute pharynx infections; the difference was statistically significant (OR = 4.18, 95% CI = 2.88–6.06, *P* = .000 < 0.01) (Fig. 3).

#### 3.4.2. The VAS scores.

**Figure 4.**
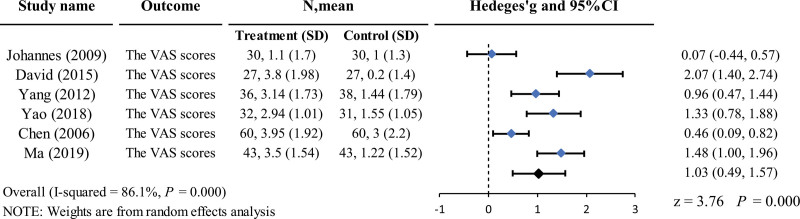
Forest plot of the VAS scores reduction. CIs = confidence intervals, SD = standard deviation.

Six studies^[21,22,30,34,39,40]^ including 457 patients reported a change in the VAS scores from the baseline. Four of these studies^[21,30,34,39]^ used antibiotics, and 2^[22,40]^ used sham acupuncture as the control group. The heterogeneity between the studies was *I*^2^ = 83.9% and *I*^2^ = 87.0%, respectively. A random-effects model was used for the meta-analysis. The results showed that acupuncture was superior to antibiotics for acute pharyngeal infection in reducing the VAS scores, and the difference was statistically significant (Hedge’s *g =* 1.30, 95% CI = 0.61–2.00, *P* = .000 < 0.01). However, the difference in VAS scores between the acupuncture and sham acupuncture groups was insignificant (Fig. 4).

#### 3.4.3. Sore throat duration.

**Figure 5.**
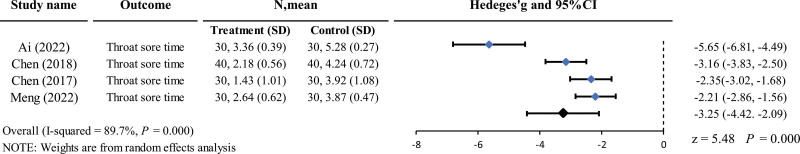
Forest plot of sore throat time reduction. CIs = confidence intervals, SD = standard deviation.

Four studies^[31,41,43,45]^ including 260 patients reported sore throat disappearance times. The heterogeneity among the studies was *I*^2^ = 89.7%; therefore, a random-effects model was selected. The results showed that acupuncture was superior to antibiotics for acute pharyngeal infection in reducing the duration of sore throat. This difference was statistically significant (Hedge’s *g =* −3.25, 95% CI = −4.42 to −2.09, *P* = .000 < 0.01) (Fig. 5).

#### 3.4.4. Laboratory indicators.

**Figure 6.**
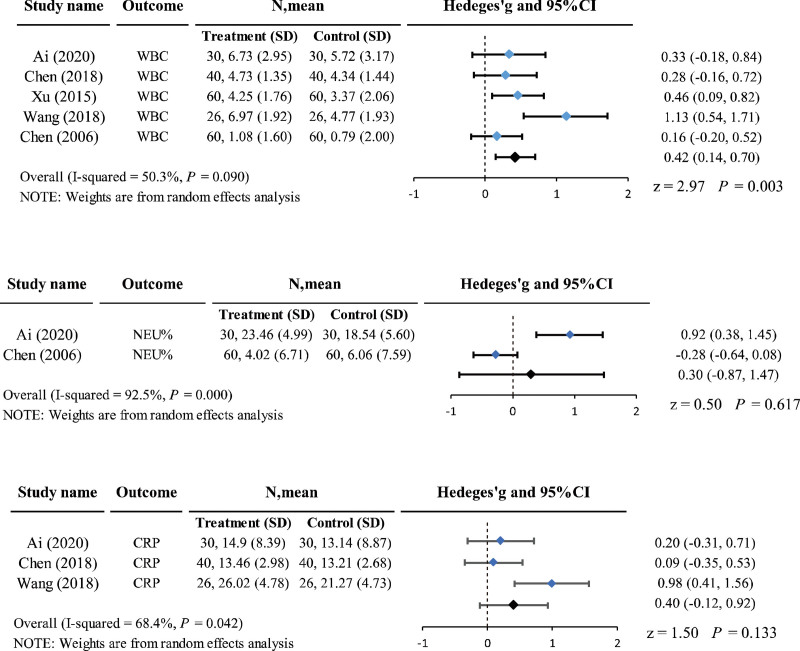
Forest plot of laboratory indicators: WBC, NEU%, CRP. CIs = confidence intervals, CRP = C-reactive protein, NEU% = neutrophil percentage, SD = standard deviation, WBCs = white blood cells.

Five studies^[30,32,36,41,45]^ reported the white blood cells (WBCs), 2 studies^[30,41]^ reported neutrophil percentage (NEU%), and 3 studies^[36,41,45]^ reported a change in the C-reactive protein (CRP) from baseline, including 432,180,192 patients. The heterogeneity between studies was *I*^2^ = 50.3%, 92.5%, and 68.4%, respectively; therefore, a random-effects model was selected. The results demonstrated that acupuncture was superior to antibiotics for acute pharyngeal infection in reducing the WBCs; the difference was statistically significant (Hedge’s *g =* 0.42, 95% CI = 0.14–0.70, *P* = .003 < 0.05). However, the differences in the modulation of NEU% and CRP levels were not significant (Fig. 6).

### 3.5. Subgroup analysis

We applied categorical and continuous variables to perform subgroup analyses, as shown in Table 3. The type of acupuncture and control group, and treatment course significantly affected the VAS scores. Auricular (*g =* 2.07, 95% CI = 1.40–2.74, *P* = .000) and bloodletting acupuncture (*g =* 1.07, 95% CI = 0.38–1.76, *P* = .002) significantly lowered the VAS scores compared to manual acupuncture (*g =* 0.51, 95% CI = −0.36 to 1.39, *P* = .248). In the control group, the antibiotics (*g =* 1.30, 95% CI = 0.61–2.00; *P* = .000) significantly lowered the VAS scores compared to sham acupuncture (*g =* 0.51, 95% CI = −0.36 to 1.39; *P* = .248). Moreover, 3, 5, and 7 days of treatment (*g =* 1.33, 95% CI = 0.78–1.88, *P* = .000), (*g =* 1.48, 95% CI = 1.00–1.96, *P* = .000), (*g =* 0.46, 95% CI = 0.09–0.82, *P* = .013) significantly lowered the VAS scores compared to treatment only once (*g =* 1.01, 95% CI = −0.04 to 2.06, *P* = .059).

**Table 3:** The subgroup analysis for the effects of acupuncture on measurement outcomes.

Variables	The response rate	The VAS scores	Throat sore time	Laboratory indicators		
				WBC (10^9/L)	NEU%	CRP (mg/L)
Age						
≥18	4.62 (2.67, 7.99)	0.85 (0.15, 1.56)	−3.65 (−5.21, −2.08)	**0.23 (−0.01, 0.48**)	0.30 (−0.87, 1.47)	0.14 (−0.20, 0.47)
<18	3.82 (2.30, 6.36)	1.41 (1.05, 1.78)	−2.21 (−2.86, −1.56)	0.75 (0.10, 1.40)	/	**0.98 (0.41, 1.56**)
Types of acupuncture						
Blood-letting	4.21 (2.82, 6.28)	1.07 (0.38, 1.76)	−3.25 (−4.42, −2.09)	0.42 (0.14, 0.70)	0.30 (−0.87, 1.47)	0.40 (−0.12, 0.92)
Manual acupuncture	3.93 (1.41, 10.98)	**0.51 (−0.36, 1.39**)	/	/	/	/
Auricular acupuncture	/	2.07 (1.40, 2.74)	/	/	/	/
Types of treatment group						
Acupuncture	3.25 (2.04, 5.18)	0.50 (0.04, 0.95)	−2.68 (−3.62, −1.74)	**0.21 (−0.07, 0.48**)	−0.28 (−0.64, 0.08)	0.09 (−0.35, 0.53)
Acupuncture + antibiotics	6.23 (3.31, 11.74)	1.58 (1.19, 1.97)	−3.96 (−7.20, −0.73	0.60 (0.18, 1.02)	**0.92 (0.38, 1.45**)	0.58 (−0.19, 1.35)
Types of control group						
Antibiotics	4.18 (2.88, 6.06)	1.30 (0.61, 2.00)	−3.25 (−4.42, −2.09)	0.42 (0.14, 0.70)	0.30 (−0.87, 1.47)	0.40 (−0.12, 0.92)
Sham acupuncture	/	**0.51 (−0.36, 1.39**)	/	/	/	/
Treatment course						
Once	/	**1.01 (−0.04, 2.06**)	/	/	/	/
3 d	4.78 (2.85, 8.00)	1.33 (0.78, 1.88)	−2.68 (−3.62, −1.74)	0.38 (0.10, 0.66)	/	/
5 d	2.65 (1.28, 5.51)	1.48 (1.00, 1.96)	−3.96 (−7.20, −0.73)	**0.33 (−0.18, 0.84**)	**0.92 (0.38, 1.45**)	0.20 (−0.31, 0.71)
7 d	5.07 (2.25, 11.44)	0.46 (0.09, 0.82)	/	**0.61 (−0.33, 1.56**)	−0.28 (−0.64, 0.08)	0.52 (−0.36, 1.39)
Sample size (n)						
<60	3.24 (1.64, 6.37)	2.07 (1.40, 2.74)	/	1.13 (0.54, 1.71)	/	**0.98 (0.41, 1.56**)
60–120	5.94 (3.07, 11.51)	0.96 (0.34, 1.58)	−3.25 (−4.42, −2.09)	**0.30 (−0.04, 0.63**)	**0.92 (0.38, 1.45**)	0.14 (−0.20, 0.47)
≥120	3.60 (1.95, 6.66)	0.46 (0.09, 0.82)	/	0.31 (0.02, 0.60)	−0.28 (−0.64, 0.08)	/

Contrary subgroup analysis data is bolded.

CRP = C-reactive protein, NEU% = neutrophil percentage, WBCs = white blood cells.

Age, treatment group type, treatment course, and sample size significantly affected the effect of acupuncture on the WBC count. Age < 18 (*g =* 0.75, 95% CI = 0.10–1.40; *P* = .024) significantly modulated the WBC counts compared to mean age ≥ 18 (*g =* 0.23, 95% CI = −0.01 to 0.48; *P* = .061) for the participants. In addition, acupuncture plus antibiotics (*g =* 0.60, 95% CI = 0.18–1.02, *P* = .006) significantly modulated the WBC counts compared to simple acupuncture (*g =* 0.21, 95% CI = −0.07 to 0.48, *P* = .146). Moreover, 3 days of treatment (*g =* 0.38, 95% CI = 0.10–0.66, *P* = .007) significantly modulated the WBC counts compared to 5 or 7 days of treatment (*g =* 0.33, 95% CI = −0.18 to 0.84, *P* = .211; (*g =* 0.61, 95% CI = −0.33 to 1.56, *P* = .204, respectively). Additionally, regarding the sample size, compared to 60 ≤ n < 120 (*g =* 0.30, 95% CI = −0.04 to 0.63, *P* = .080), n < 60 (*g =* 1.13, 95% CI = 0.54–1.71, *P* = .000) or n ≥ 120 (*g =* 0.31, 95% CI = 0.02–0.60, *P* = .039) significantly modulated the WBC counts.

The treatment group type, treatment course, and sample size significantly affected the NEU%. Acupuncture plus antibiotics (*g =* 0.92, 95% CI = 0.38–1.45, *P* = .001) significantly modulated the NEU% compared to simple acupuncture (*g =* −0.28, 95% CI = −0.64 to 0.08, *P* = .123). Moreover, 5 days of treatment (*g =* 0.92, 95% CI = 0.38–1.45, *P* = .001) significantly modulated the NEU% compared to 7 days of treatment (*g =* −0.28, 95% CI = −0.64 to 0.08, *P* = .123). Additionally, compared to n ≥ 120 (*g =* −0.28, 95% CI = −0.64 to −0.08, *P* = .123), RCTs with 60 ≤ n < 120 (*g =* 0.92, 95% CI = 0.38–1.45, *P* = .001) significantly modulated the NEU%. Age and sample size significantly modulated the effect of acupuncture on the CRP levels. Mean age < 18 (*g =* 0.98, 95% CI = 0.41–1.56, *P* = .001) significantly modulated CRP compared to mean age ≥ 18 (*g =* 0.14, 95% CI = −0.20 to 0.47; *P* = .422) for the participants. Moreover, compared to 60 ≤ n < 120 (*g =* 0.14, 95% CI = −0.20 to 0.47; *P* = .422), n < 60 (*g =* 0.98, 95% CI = 0.41–1.56, *P* = .001) significantly modulated the CRP.

### 3.6. Meta-regression

In the meta-regression (Table 4), the treatment group type was a significant covariate (β = 0.2598, 95% CI = −0.9280 to 1.4477; *P* = .030) for the VAS scores in the regression analyses. However, age, acupuncture type, control group type, treatment course, and sample size did not significantly affect the other outcomes (*P* > .05).

**Table 4:** Meta-regression to predict acupuncture effects on measurement outcomes.

Variables	The response rate	The VAS scores	The duration of the sore throat	Laboratory indicators	
				WBC (10^9/L)	CRP (mg/L)
Age	−0.8033 (−2.2756, 0.6689)	0.5529 (−1.2157, 2.3216)	1.4621 (−6.8786, 9.8028)	0.4247 (−0.3280, 1.1774)	0.8478 (−3.4732, 5.1688)
Types of acupuncture	0.1185 (−1.3720, 1.6091)	0.2598 (−0.9280, 1.4477)	/	/	/
Types of treatment group	1.0169 (−0.1100, 2.1438)	1.0963 (0.1775, 2.1050)	−1.2490 (−8.5715, 6.0735)	0.3618 (−0.4800, 1.2035)	0.4924 (−7.8618, 8.8467)
Types of control group	/	−0.7845 (−2.3729, 0.8039)	/	/	/
Treatment course	−0.1109 (−0.4536, 0.2317)	−0.0417 (−0.4271, 0.3438)	−0.6245 (−4.2857, 3.0367)	0.0453 (−0.2665, 0.3571)	0.1569 (−4.7678, 5.0816)
Sample size	−0.2341 (−1.0458, 0.5774)	−0.7811 (−1.9242, 0.3620)	/	−0.2922 (−0.8467, 0.2622)	0.8478 (−5.1688, 3.4732)

CRP = C-reactive protein, WBCs = white blood cells.

### 3.7. Publication bias

As the clinical response rate was a dichotomous variable with low heterogeneity (*I*^2^ = 0.0%), we used Harbord’s test to test for publication bias, which maintains the power of Egger’s test while reducing the false-positive rate.^[28]^ Harbor’s test showed *P* = .389 > 0.05, thereby indicating no significant publication bias. Egger’s test was used to assess publication bias for other continuous variable outcome indicators. The results revealed that the VAS scores (*P* = .157 > 0.05), sore throat duration (*P* = .072 > 0.05), WBC count (*P* = .206 > 0.05), and CRP levels (*P* = .270 > 0.05) indicated no significant publication bias. (Table 5)

**Table 5:** Publication bias.

Outcomes	Coef.	Std. Err.	*t*	*P*> *t*	95% Conf. Interval	
**The response rate**						
Number of studies = 19					Root MSE = 0.8927	
Z/sqrt (V)						
Sqrt (V)	0.782254	0.622053	1.26	0.226	−0.5301629	2.09467
Bias	0.77307	0.874199	0.88	0.389	−1.071329	2.617469
**The VAS scores**						
Number of studies = 6					Root MSE = 2.26	
Std_Eff						
Slope	−1.20702	1.236164	−0.98	0.384	−4.639162	2.225122
Bias	8.720992	5.014076	1.74	0.157	−5.200316	22.6423
**Throat sore time**						
Number of studies = 4					Root MSE = 1.424	
Std_Eff						
Slope	1.553303	1.286633	1.21	0.351	−3.982633	7.089238
Bias	−12.20248	3.475761	−3.51	0.072	−27.15748	2.752514
**WBC**						
Number of studies = 5					Root MSE = 1.2	
Std_Eff						
Slope	−0.616091	0.636773	−0.97	0.405	−2.642587	1.410405
Bias	4.686476	2.915348	1.61	0.206	−4.591462	13.96441
**CRP**						
Number of studies = 3					Root MSE = 1.035	
Std_Eff						
Slope	−2.630656	1.351946	−1.95	0.302	−19.80876	14.54745
Bias	11.78077	5.31656	2.22	0.27	−55.77253	79.33406

CRP = C-reactive protein, MSE means square error, WBCs = white blood cells.

## 4. Discussion

This is the first meta-analysis of acupuncture for acute pharyngeal infections. In total, 1701 patients were included in 19 RCTs of acupuncture therapy for acute pharyngeal infection. Seventeen RCTs compared acupuncture therapy with antibiotics and 2 studies compared acupuncture therapy with sham acupuncture. Antibiotics, which are considered the first-line treatment for pharyngitis, reduce the average symptom duration and prevent suppurative complications.^[2]^ Therefore, comparing acupuncture therapy with antibiotics is meaningful as it can determine whether acupuncture is efficacious.

Because we rigorously screened the RCTs based on the eligibility criteria of this study, the quality assessment results of the 19 RCTs did not demonstrate a high risk of bias in terms of random sequences and allocation concealment. In terms of the blinding of the outcome assessment, one study was at high risk because the outcome assessors were not blinded.^[21]^ Notably, blinding acupuncturists and patients is challenging owing to the specific nature of acupuncture treatment. Therefore, the risks in all the studies regarding the blinding of participants and personnel were unclear.

We used Hedges’ *g* value to accurately estimate the overall effect size, which can eliminate this bias for small sample sizes.^[46]^ The meta-analysis revealed that acupuncture improved the clinical response rates and reduced the VAS scores, sore throat duration, and WBC count compared with antibiotics. However, the differences in the NEU% and CRP levels were not statistically significant. We suggest that this could be attributed to the low number of RCTs regulating the NEU% and CRP and the different acupuncture point selections and manipulations. In addition, 2 studies used sham acupuncture as a control group; no significant difference was observed between acupuncture and sham acupuncture in VAS score reduction. Therefore, the placebo effects of acupuncture remain unclear. Despite the variety of sham acupuncture methods,^[47]^ none of them are considered appropriate sham control for indistinguishability and physiological inertness.^[48]^

Sensitivity analyses were conducted to examine the stability of the results, which revealed that the results remained stable after eliminating each data point (File S1–S6, Supplemental Digital Content, http://links.lww.com/MD/J183). In addition, subgroup analyses were performed to explore the effects of other potentially confounding variables. Six subgroups were established according to a preliminary protocol. The age subgroup demonstrated that the mean age < 18 significantly modulated the WBC counts and CRP levels compared with the mean age ≥ 18 groups. Therefore, we speculate that acupuncture has a superior effect on the regulation of leukocyte and CRP levels in minors than in adults. The acupuncture subgroups demonstrated that RCTs using auricular and bloodletting acupuncture significantly lowered the VAS scores compared to RCTs using manual acupuncture. This finding indicates that bloodletting and auricular acupuncture are superior to manual analgesia for acute pharyngeal infections. The treatment types of the subgroups showed that acupuncture plus antibiotics significantly modulated the WBC count and CRP levels compared with simple acupuncture. The control group demonstrated that RCTs using sham acupuncture did not significantly reduce the VAS scores. We believe that this may be related to the method of sham acupuncture implementation, and that some sham acupuncture methods may be as effective as acupuncture.^[49]^ In the treatment course subgroup, 3, 5, and 7 days of treatment resulted in significantly lower VAS scores than those treated only once. This suggests that regular acupuncture is more effective than a single acupuncture session for analgesia in patients with acute pharyngeal infections. Moreover, RCTs with 3 days modulated the WBC counts compared to RCTs with 5 or 7 days, and RCTs with 5 days significantly modulated the NEU% compared to RCTs with 7 days. This suggests that acupuncture may have a better short-term regulatory effect on the WBC count and NEU%. The sample subgroup size showed that compared to RCTs with 60 ≤ n < 120, RCTs with n < 60 or n ≥ 120 significantly modulated the WBC counts, compared to RCTs with n ≥ 120, RCTs with 60 ≤ n < 120 significantly modulated the NEU%, and compared to RCTs with 60 ≤ n < 120, RCTs with n < 60 significantly modulated the CRP levels. A clear association between the sample size and the volatility of laboratory outcomes was not observed.

However, heterogeneity existed in all the outcome measures except for the clinical response rate. Although we performed subgroup and sensitivity analyses, we could not directly determine the cause of heterogeneity. Therefore, we conducted a meta-regression analysis to determine the principal risk factors of heterogeneity. Meta-regression analysis revealed significant relationships between the treatment groups and VAS scores, which could have significantly influenced heterogeneity. However, additional factors that significantly impact other outcomes have not been identified, which results in challenges in identifying the sources of their heterogeneity. We suggest that clinical heterogeneity is the leading risk factor associated with the other outcomes. Owing to the specific nature of acupuncture techniques, clinical heterogeneity, such as the use of different acupoints, piercing at different depths, and other manipulation techniques, cannot be avoided.

Regarding the safety analysis, no AEs of acupuncture were reported in the 19 studies. In comparison, 4 studies reported AEs of antibiotics, including allergy, rash, itchy skin, high fever and chills, diarrhea, nausea, and gastrointestinal symptoms.^[34,36,38,45]^ Compared with antibiotics, acupuncture resulted in fewer AEs and higher safety.

The treatment acupoints in the included studies were mainly in the meridians of the lung and large intestine. Five studies used *He Gu* (LI 4) and 4 used *Qu Chi* (LI 11), which were the most frequent occurrence in the large intestine meridian. The most frequently occurring acupuncture point in the lung meridian is *Shao Shang* (LU 11). In addition, 5 studies selected local acupuncture in the throat for acute pharyngeal infections. LI 4 is located on the back of the hand, between the first and second metacarpal bones, at the midpoint of the radial side of the second metacarpal; LI 11 is located on the lateral side of the elbow, at the center of the line between the *Chi Ze* (LU 5) and the lateral epicondyle of the humerus. Some studies have demonstrated that LI 4 and LI 11 stimulation can produce both hypothermia and analgesia, which may be appropriate treatments for fever and pain syndromes.^[50]^ LU 11 is located on the radial side of the finger, at the end of the thumb, and 0.1 inches above the corner of the nail base. A previous study demonstrated that needle bloodletting at LU 11 improved the symptoms of pneumonia.^[51]^ Another study demonstrated that acupuncture at LU 11 blocked the infiltration of CD4^+^ T cells and inflammatory cells to regulate inflammation.^[52]^ In addition, several studies have confirmed the local antinociceptive effect of acupuncture, thereby supporting local acupuncture in the throat.^[53]^

As the mechanism of action of acupuncture for acute pharyngeal infections remains unclear, we summarized and hypothesized a possible mechanism to provide a basis for future research. Currently, the pathogenesis of acute pharyngeal diseases mainly involves an inflammatory response of the pharynx caused by viruses and bacteria. Several studies have demonstrated that acupuncture positively regulates the human immune system. First, the anti-inflammatory effects of acupuncture involve innate immune cells regulation Acupuncture exerts anti-inflammatory effects by downregulating pro-inflammatory M1 macrophages, upregulating anti-inflammatory M2 macrophages, and regulating the secretion of related cytokines.^[54–56]^ Acupuncture enhances natural killer cell (NK) activity. After acupuncture stimulation, the function of immune cells (CD16+, CD56, and interferon-gamma levels), which are closely related to NK cell activity, was strengthened in the peripheral blood of healthy volunteers.^[57]^ Acupuncture can activate mast cells by activating transient receptor potential vanilloid (TRPV)1 and TRPV2,^[58]^ which promote degranulation and the release of histamine and adenosine to initiate neuroimmune regulation.^[59]^ Second, the anti-inflammatory effects of acupuncture involve the regulation of adaptive immune cells. Acupuncture therapy can balance T-lymphocyte subpopulations and reduce cytokine secretion, which is closely related to the inflammatory response.^[60]^ Third, the anti-inflammatory effect of acupuncture involves the regulation of the neuroimmune system. Acupuncture inhibits the release of substance P (SP), calcitonin gene-related peptide (CGRP), and other neuropeptides from the dorsal root ganglion. It inhibits TRPV1 and its downstream signaling pathways by reducing peripheral neurogenic inflammation and transmission of pain signals to the central nervous system.^[61]^ Some studies have demonstrated that acupuncture suppresses inflammation by activating neurons in the brainstem.^[62]^ These theories provide a theoretical basis for evaluating the effectiveness of acupuncture for the treatment of pharyngeal infections.

### 4.1. Limitation

First, the small sample sizes of some studies could have increased the potential of biased results in our study. Second, owing to the specificity of acupuncture therapy, different acupuncturists follow various treatment plans. Third, only English and Chinese languages were selected as the literature retrieval languages, which increased the risk of selection bias. Fourth, there was no evidence to guide the choice of acupuncture manipulation, depth, or time. Finally, based on the available data, the effective boundary of different antibiotics for acute pharyngeal infections cannot be defined. The results obtained from this meta-analysis show that acupuncture treatment is superior to antibiotics in improving the clinical response rates, reducing the VAS scores, sore throat duration, and WBC regulation. However, a placebo effect could not be excluded.

### 4.2. Future prospects

First, it is essential to interpret the mechanisms underlying the effects of acupuncture in acute pharyngeal infections from a pathophysiological perspective. Fundamental studies on acupuncture therapy for acute pharyngeal infections should be conducted to explore the potential use of acupuncture in treating acute pharyngeal infections. Second, additional control groups for acupuncture and sham acupuncture should be established to confirm the placebo effect. Third, studies with large sample sizes are warranted to prevent the impact of small sample sizes and further improve the quality of evidence. Finally, standardized acupuncture treatments should be used in future studies, including uniform acupoints, acupuncture depth, manipulation technique, and treatment time.

## 5. Conclusion

Our study supports the idea that acupuncture can be considered as an auxiliary treatment for acute pharyngeal infections. Acupuncture therapy for acute pharyngeal infections is safe and its response rate is superior to that of antibiotics. Acupuncture has demonstrated positive outcomes for alleviating sore throat symptoms, reducing sore throat duration, and improving immune inflammation index. Nevertheless, owing to the limitations of this study, our conclusions should be interpreted with caution. Future efforts warrant more high-quality trials to improve the methodology and reporting quality.

## Acknowledgments

The authors acknowledge all authors of the original studies used. Additionally, we would like to thank *Editage* (www.editage.cn) for English language editing.

## Author contributions

**Conceptualization:** Yang Cui, Xiangyue Meng.

**Formal analysis:** Delong Wang.

**Methodology:** Jiantao Yin.

**Software:** Xinyu Zhou, Yu Cao.

**Writing – original draft:** Shuo Zhang.

**Writing – review & editing:** Quan Li, Hongna Yin.

## Supplementary Material


