# Supplementary material for: Efficacy of acupuncture on acute pharynx infections: A systematic review and meta-analysis
Source: Medicine (Baltimore). 2023 Jun 23;102(25):e34124. doi: 10.1097/MD.0000000000034124 (PMC10289600; doi:10.1097/MD.0000000000034124)

S1. The sensitivity analyses of response rate

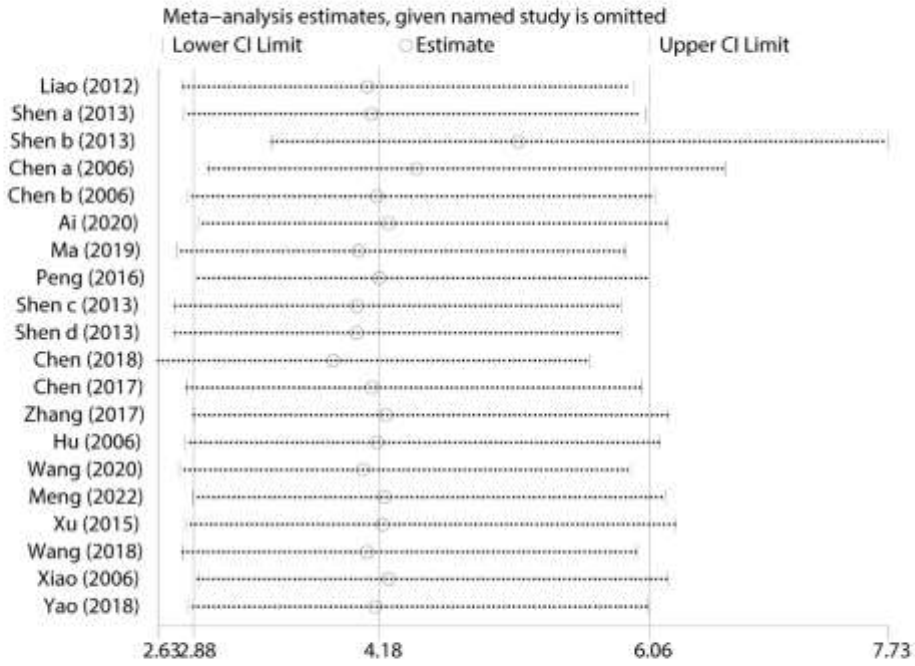

S2. The sensitivity analyses of VAS scores

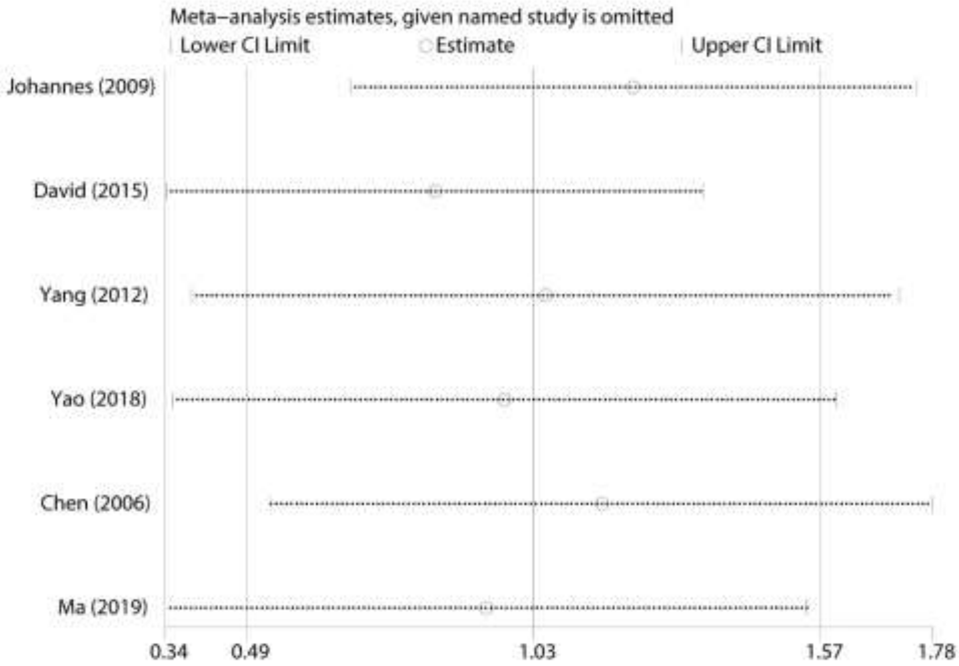

S3. The sensitivity analyses of sore throat time

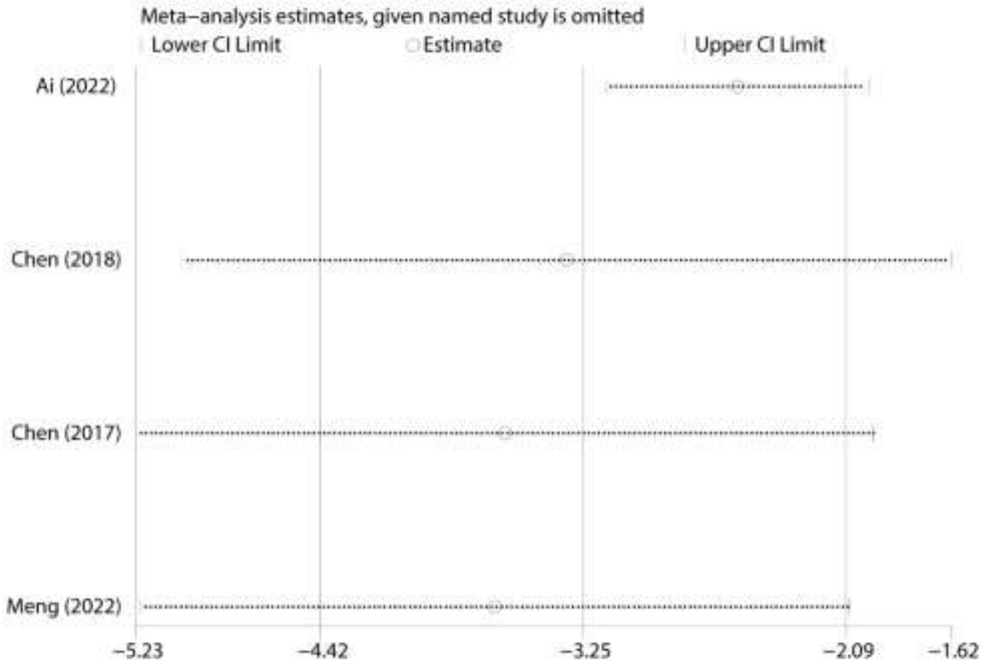

S4. The sensitivity analyses of WBC

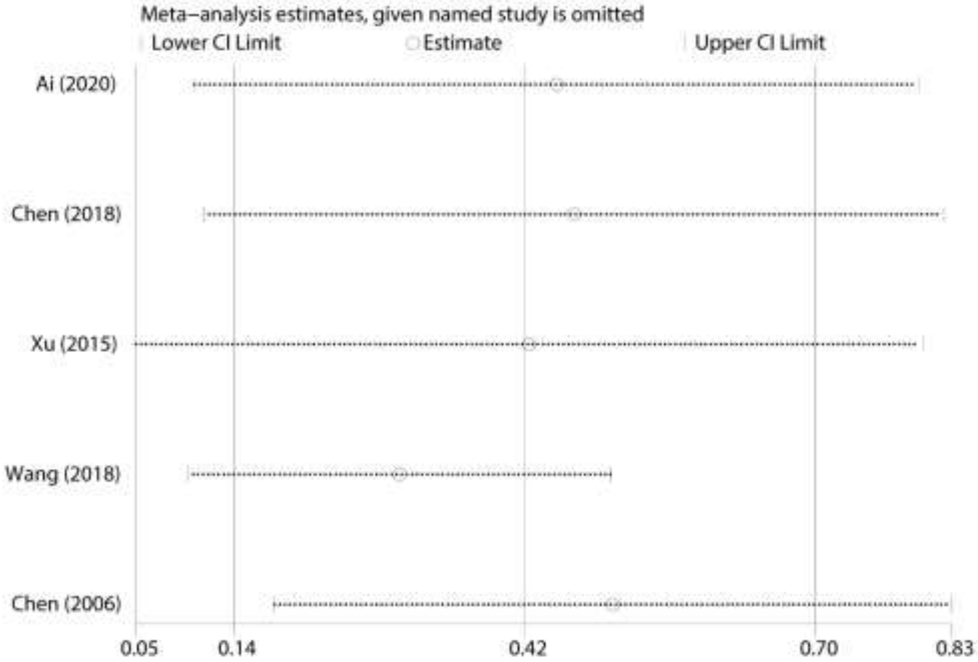

S5. The sensitivity analyses of NEU%

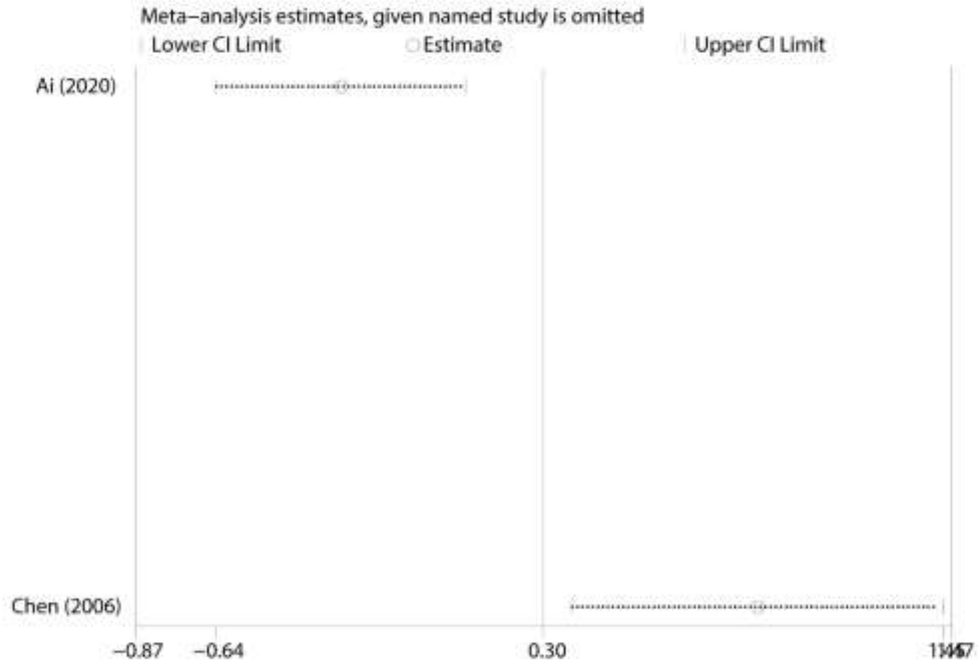

S6. The sensitivity analyses of CRP

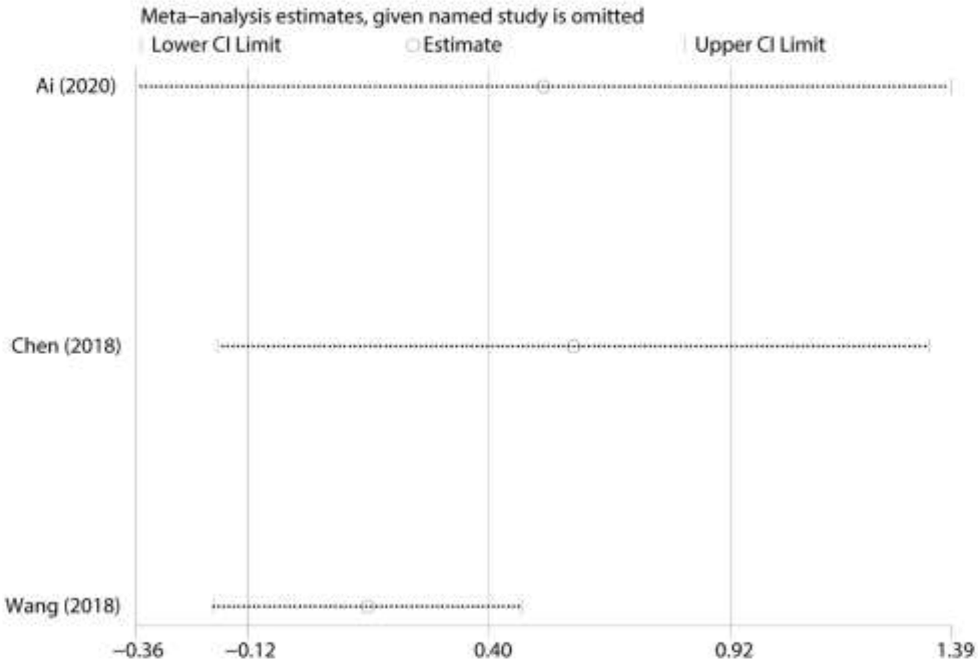

Supplement: Supplementary file 1 [file medi-102-e34124-s001.pdf]
